# Endovascular repair of complex aortic aneurysms: comparison between surgeon-modified endografts and commercial branched devices in a high-complexity center

**DOI:** 10.47487/apcyccv.v6i4.544

**Published:** 2025-12-29

**Authors:** Roger Conde Moncada, Luis Mariano Ferreira, Ricardo La Mura, Oscar Dávila, W. Samir Cubas

**Affiliations:** 1 Servicio de Cirugía Cardiovascular y Endovascular, Instituto Cardiovascular del Perú, Lima, Perú. Servicio de Cirugía Cardiovascular y Endovascular Instituto Cardiovascular del Perú Lima Perú; 2 Servicio de Cirugía Vascular y Endovascular, Clínica Sagrada Familia, Buenos Aires, Argentina. Servicio de Cirugía Vascular y Endovascular Clínica Sagrada Familia Buenos Aires Argentina; 3 Departamento de Cirugía Cardiovascular, London Health Sciences Centre, London, Canadá. Departamento de Cirugía Cardiovascular London Health Sciences Centre London Canadá

**Keywords:** Aneurisma de la Aorta Toracoabdominal, Reparación Endovascular de Aneurismas, Mortalidad Hospitalaria, Aortic Aneurysm Thoracoabdominal, Endovascular Aneurysm Repair, Hospital Mortality

## Abstract

**Objetivo.:**

El tratamiento endovascular de los aneurismas aórticos toracoabdominales y paraviscerales complejos, con el desarrollo de nuevos dispositivos, representa uno de los aspectos más desafiantes de la cirugía endovascular. El objetivo de este estudio fue describir la mortalidad perioperatoria a 30 días, las complicaciones mayores posoperatorias y las reintervenciones de los pacientes tratados por aneurismas aórticos complejos mediante dispositivos de endoprótesis ramificadas *off-the-shelf* (t-BRANCH) y *Physician Modified Endografts* (PMEGs).

**Materiales y métodos.:**

El presente trabajo es un estudio observacional, retrospectivo y unicéntrico sobre una base de datos prospectivamente recolectada de cada paciente tratado por aneurisma aórtico complejo registrado en la historia clínica de nuestro centro aórtico de referencia, entre enero de 2020 y diciembre de 2024.

**Resultados.:**

Se analizaron 51 pacientes con una media de edad de 69,6 ± 10,3 años, siendo varones el 90,2%. El diámetro medio del aneurisma fue de 66,1 ± 15,2 mm. La mortalidad global fue del 9,8%, siendo la mortalidad intrahospitalria temprana en t-BRANCH del 23,1% en comparación con PMEGs del 5,3% (p=0,0977). Dentro de los predictores de mortalidad intrahospitalaria se encontraron el estado físico según la clasificación del estado físico de la Sociedad Americana de Anestesiólogos (ASA) IV (OR = 11.98; IC95%: 1.46-98.7; p = 0.022) y el antecedente de accidente cerebrovascular (ACV) (OR = 13.07; IC95%: 1.06-161.5; p = 0.043).

**Conclusiones.:**

La reparación endovascular de aneurismas complejos de aorta mediante endoprótesis con PMEGs y dispositivos t-BRANCH muestra resultados favorables con respecto a la mortalidad y las complicaciones mayores posoperatorias, asociadas a una baja tasa de reintervenciones.

## Abstract (es)

**Objective.:**

Endovascular treatment of complex thoracoabdominal and paravisceral aortic aneurysms represents one of the most demanding challenges in endovascular surgery, driven by the development of new devices. This study aimed to describe the 30-day perioperative mortality, major postoperative complications, and reinterventions in patients treated for complex aortic aneurysms using off-the-shelf branched endografts (t-BRANCH) and Physician-Modified Endografts (PMEGs).

**Materials and Methods.:**

This was a single-center, retrospective, observational study based on a prospectively collected database including all patients treated for complex aortic aneurysms and recorded in their medical records at our reference aortic center between January 2020 and December 2024.

**Results.:**

A total of 51 patients were analyzed, with a mean age of 69.6 ± 10.3 years; 90.2% were male. The mean aneurysm diameter was 66.1 ± 15.2 mm. Overall mortality was 9.8%, with early in-hospital mortality of 23.1% in the T-Branch group compared to 5.3% in the PMEG group (p = 0.0977). Predictors of in-hospital mortality included an American Society of Anesthesiologists (ASA) physical status classification of IV (OR = 11.98; 95% CI: 1.46-98.7; p = 0.022) and a history of stroke (OR = 13.07; 95% CI: 1.06-161.5; p = 0.043).

**Conclusions.:**

Endovascular repair of complex aortic aneurysms using PMEGs and t-BRANCH devices shows favorable results with respect to mortality and major postoperative complications associated with a low rate of reinterventions.

## Introduction

Endovascular treatment of complex thoracoabdominal and paravisceral aortic aneurysms represents one of the most demanding challenges in endovascular surgery. The development of new devices for their appropriate management, such as fenestrated and branched endografts, has enabled successful treatment of patients with complex anatomy, multiple comorbidities, or contraindications to open surgery, achieving acceptable perioperative morbidity and mortality outcomes ^1^^,^^2^.

The availability of off-the-shelf branched endografts (t-BRANCH) has proven to be an effective and readily accessible alternative, owing to their standardised configurations, for the urgent or elective treatment of thoracoabdominal aneurysms ^3^. Conversely, in the absence of commercially available custom-made fenestrated devices (Custom Made Devices, CMDs), which typically require manufacturing times of approximately three months, some centres have developed advanced endovascular techniques such as physician-modified endografts (PMEGs) ^4^^-^^7^.

Globally, the study by Robaldo *et al.* showed that 34.4% of centres perform fewer than ten PMEG cases per year. Italy leads in utilisation, followed by the United States, Canada, and Austria; in Latin America, Brazil ranks first, followed by Colombia and Argentina. These findings highlight the limited availability of this technology in the region and underscore the importance of reporting institutional experiences ^8^.

The aim of this study was to describe 30-day perioperative mortality, major postoperative complications, and reinterventions among patients treated for complex aortic aneurysms using t-BRANCH devices and PMEGs, as well as the factors associated with these outcomes.

## Materials and methods

### Design and study population

This is an observational, retrospective, single-centre study based on a prospectively collected database including all patients treated for complex aortic aneurysms recorded in their medical records between January 2020 and December 2024 at our reference aortic centre, Clínica Sagrada Familia, located in Buenos Aires, Argentina. Patients treated for complex thoracoabdominal or paravisceral aneurysms using t-BRANCH or fenestrated endografts employing the PMEGs technique were included. Exclusion criteria comprised aortic repairs not involving fenestrated or branched endografts, hybrid surgeries, procedures converted to open surgery, and patients with incomplete follow-up (less than 30 days without available clinical or imaging data).

### Data collection and variable assessment

Clinical, anatomical, technical, and perioperative data were collected retrospectively from electronic medical records and diagnostic imaging. Subsequently, a manual review was performed to validate the completeness and consistency of the collected information. Data were coded and organised in a structured database using Microsoft Excel and R software (Microsoft Corp., Redmond, WA, USA).

The following variables were considered in the present study: demographic data (age and sex); comorbidities (hypertension, diabetes mellitus, smoking status, dyslipidaemia, history of stroke, obesity, coronary artery disease, chronic obstructive pulmonary disease, among others); anatomical characteristics of the aorta (diameter, location, and extent of the aneurysm); type of device used (t-BRANCH or PMEGs); technical parameters (number of revascularised vessels); and clinical outcomes.

Perioperative mortality was also assessed and defined as any death occurring during the surgical procedure, within the first 30 days after the intervention, or during the index hospitalisation, regardless of the time elapsed since surgery or hospital discharge. Postoperative cardiovascular complications were defined as adverse postoperative events that significantly compromised the patient’s clinical status, including spinal cord ischaemia, stroke, myocardial infarction, renal failure, heart failure, and cardiogenic shock. Spinal cord ischaemia was defined as a new motor or sensory deficit occurring during or after the endovascular procedure. Reinterventions were defined as any additional endovascular procedure required to correct procedure-related complications, maintain patency of the endograft and its branches or fenestrations along their entire length, or treat new lesions identified during follow-up.

### Procedure description

All procedures were performed in the catheterisation laboratory under general anaesthesia and involved invasive monitoring, including a radial arterial line and a central jugular venous catheter. Vascular access was obtained through surgical exposure, with an inguinal incision and layered tissue dissection to expose the common femoral artery, followed by appropriate vascular control using vessel loops. The same procedure was performed on the contralateral side. When the left subclavian artery was used, a left infraclavicular approach was employed. Percutaneous access was not used in any case. Preoperative planning included high-resolution computed tomographic angiography with centreline reconstructions and multiplanar analyses using Horos software (Horos Project, version 4.0.2) to assess aortic morphology and visceral vessel anatomy.

All interventions were guided by fusion imaging (Vessel Navigator, Azurion/Alura Xper FD20, Philips Healthcare) and intraoperative cone-beam computed tomography (Xpert-CT, Philips), allowing precise endograft deployment and accurate identification of target vessels.

In all cases, the type of endograft was selected after multidisciplinary discussion. In the t-BRANCH group, preloaded multibranched off-the-shelf endografts were used without modification. In contrast, in the PMEG group, the selected endografts were manually modified by the surgical team. Fenestrations were created intraoperatively and reinforced with radiopaque snares sutured using 5-0 polyester (Ethibond), followed by the creation of constraining ties; the devices were then re-sheathed for subsequent implantation in the patient.

With regard to the stents used to connect the branches or fenestrations to the visceral arteries (celiac trunk, superior mesenteric artery, and renal arteries), no single brand was specified; selection was based on operator preference.

After completion of each procedure, a final completion angiography was performed in all cases to confirm the correct positioning of the endograft, its extensions, and the stents deployed in the corresponding visceral vessels. The absence of type I or type III endoleaks was also assessed and addressed immediately when detected. Following the procedure, patients were transferred to the coronary care unit for postoperative management.

Patients underwent computed tomography angiography (CTA) prior to discharge to verify aneurysm exclusion, device integrity, and visceral vessel patency. In patients with renal insufficiency, non-contrast CT was performed. For follow-up, scheduled imaging was obtained at 6 and 12 months, followed by annual surveillance thereafter.

### Ethical aspects

The present study was approved by the institutional ethics committee. As it did not involve additional interventions or direct contact with patients and used only anonymised clinical records, informed consent was not required. Confidentiality and ethical handling of the data were ensured at all times.

### Statistical analysis

Quantitative variables were expressed as mean ± standard deviation or median (interquartile range), according to their distribution, while categorical variables were expressed as absolute frequencies and percentages. The chi-square test or Fisher’s exact test was used to compare proportions, and the Student’s t test or the Mann-Whitney U test was applied for continuous variables. A p-value <0.05 was considered statistically significant. To analyse predictors of adverse events, a multivariable logistic regression model was used, including variables with p <0.10 in the univariable analysis. 

Statistical analyses were performed using R software and Microsoft Excel (Microsoft Corp., Redmond, WA, USA).

## Results

Fifty-one patients who underwent endovascular repair of complex thoracoabdominal or paravisceral aortic aneurysms between January 2020 and December 2024 were included. Of these, 38 (74.5%) were treated with PMEGs and 13 (25.5%) with t-BRANCH. The mean age was 69.6 ± 10.3 years, and most patients were male (90.2%).

The most frequent comorbidities were hypertension (90.2%), smoking (76.5%), chronic obstructive pulmonary disease (78.4%), dyslipidaemia (52.9%), and coronary artery disease (47.1%). Diabetes mellitus was present in 27.5%, and a prior history of stroke in 2.0%. According to the American Society of Anesthesiologists (ASA) physical status classification, 70.6% were ASA III and 29.4% were ASA IV (Table 1).

**Table 1:** Baseline clinical characteristics of the study population.

Variable	Overall (n=51)	PMEGs (n=38)	T-BRANCH (n=13)
Age (years), mean ± SD	69.63 ± 10.32	70.45 ± 8.2	67.23 ± 11.4
Male sex	46 (90.2%)	33 (86.8%)	13 (100%)
Hypertension	46 (90.2%)	35 (92.1%)	11 (84.6%)
Smoking	39 (76.47%)	30 (78.95%)	9 (69.23%)
Dyslipidaemia	27 (52.9%)	20 (52.6%)	7 (53.8%)
Diabetes mellitus	14 (27.5%)	3 (7.9%)	11 (84.6%)
History of cancer	36 (70.6%)	26 (68.4%)	10 (76.9%)
Prior stroke	1 (2.0%)	0 (0%)	1 (7.7%)
Obesity	38 (74.5%)	31 (81.6%)	7 (53.8%)
Ischaemic coronary heart disease	24 (47.1%)	18 (47.4%)	6 (46.2%)
COPD	40 (78.4%)	29 (76.3%)	11 (84.6%)
Prior EVAR	11 (21.6%)	11 (28.9%)	0 (0%)
Prior TEVAR	7 (13.7%)	6 (15.8%)	1 (7.7%)
Peripheral arterial disease	5 (9.8%)	3 (7.9%)	2 (15.38%)
Prior ruptured aneurysm	3 (5.9%)	1 (2.6%)	2 (15.38%)
History of Marfan syndrome	2 (3.92%)	1 (2.63%)	1 (7.69%)
ASA III	36 (70.6%)	29 (76.3%)	7 (53.8%)
ASA IV	15 (29.4%)	9 (23.7%)	6 (46.2%)
Shaggy aorta	2 (%)	2 (%)	0 (0%)
In-hospital mortality	5 (9.8%)	3 (7.89%)	2 (15.38%)
Postoperative stroke	4 (7.8%)	2 (5.52%)	2 (15.23%)
Paraplegia	0 (0%)	0 (0%)	0 (0%)
Renal failure	33 (64.7%)	27 (71.1%)	6 (46.2%)
Aneurysm diameter (mm), mean ± SD	66.1 ± 15.2	65.11 ± 13.6	71.0 ± 19.4
Length of hospital stay (days), mean ± SD	14.6 ± 3.2	14.03 ± 2.6	15.54 ± 3.6

PMEGs: Physician-Modified Endografts. t-BRANCH: off-the-shelf branched endografts. COPD: chronic obstructive pulmonary disease. EVAR: endovascular abdominal aortic aneurysm repair. TEVAR: thoracic endovascular aortic repair. History of cancer: prior malignancy treated with survival >5 years. ASA: American Society of Anesthesiologists physical status classification. SD: standard deviation.

Seven patients (13.7%) had a history of prior thoracic endovascular aortic repair (TEVAR) and 11 (35.3%) had undergone previous endovascular abdominal aortic aneurysm repair (EVAR). In addition, 5.9% presented with ruptured aneurysms at admission (Table 1).

Before intervention, the mean aneurysm diameter was 66.1 ± 15.2 mm. According to anatomical classification, juxtarenal aneurysms were the most frequent (45.1%), followed by Crawford type IV (15.7%) and pararenal aneurysms (9.8%) (Table 2). Only two patients had Marfan syndrome, and two had aortic dissection.

**Table 2:** Aneurysm type.

Variable	Frequency (n)	Percentage (%)
Thoracoabdominal aneurysms		
Crawford type I	1	2.0
Crawford type II	3	5.9
Crawford type III	2	3.9
Complex abdominal aneurysms		
Crawford type IV	8	15.7
Suprarenal saccular	1	2.0
Infrarenal + type IA endoleak	4	7.8
Juxtarenal	23	45.1
Juxtarenal + type IA endoleak	2	3.9

Overall, in-hospital mortality was 9.8% Table 1). When comparing endograft types, early in-hospital mortality was higher in the t-BRANCH group (23.1%) compared with PMEGs (5.3%), although this difference did not reach statistical significance (p = 0.0977). Postoperative cardiovascular complications occurred in 7.9% of PMEG-treated patients and 7.7% of those treated with t-BRANCH, with no significant difference (p = 1.000) (Table 3). Only four patients experienced a postoperative stroke, and no cases of paraplegia were recorded (Table 1).

**Table 3:** Comparison between PMEGs and t-BRANCH

Variable	PMEGs (n = 38)	t-BRANCH (n = 13)	p-value
Postoperative cardiovascular complications	3 (7.9%)	1 (7.7%)	1.000
Early reintervention (<30 days)	2 (5.3%)	1 (7.7%)	1.000
Early in-hospital mortality	2 (5.3%)	3 (23.1%)	0.098
Length of hospital stay (days), mean ± SD	14.03 ± 6.97	15.54 ± 3.72	0.457

t-BRANCH: off-the-shelf branched endografts. PMEGs: Physician-Modified Endografts. SD: standard deviation.

Early reinterventions occurred in 5.3% of PMEG-treated patients and 7.7% of those treated with t-BRANCH (p = 1.000). There was no significant difference in mean hospital length of stay between PMEGs (14.0 ± 26.9 days) and t-BRANCH (15.5 ± 33.7 days) (Table 3).

To identify predictors of mortality, univariable analysis was performed (Table 4), identifying ASA IV physical status (odds ratio [OR] = 11.98; 95% confidence interval [CI]: 1.46-98.7; p = 0.022) and a history of stroke (OR = 13.07; 95% CI: 1.06-161.5; p = 0.043) as strong predictors of in-hospital mortality. In multivariable adjusted analysis, only a history of stroke remained independently associated with mortality (OR = 30.48; 95% CI: 1.58-1390.36; p = 0.034) (Table 5). Coronary artery disease and renal dysfunction were notable but did not reach statistical significance (p = 0.120 and p = 0.144, respectively). No associations were found between late complications and aneurysm diameter, renal dysfunction, or coronary artery disease.

**Table 4:** Univariable analysis of clinical characteristics predicting in-hospital mortality.

Variable	Frequency n (%)	OR (95% CI)	p-value
ASA class IV (vs. ASA III)	14 (27.5%)	11.98 (1.46-98.7)	0.022
History of stroke (vs. no stroke)	6 (11.8%)	13.07 (1.06-161.5)	0.043
ICHD (vs. none)	10 (19.6%)	4.60 (0.68-31.1)	0.120
eGFR <60 mL/min/1.73 m² (vs. ≥60)	8 (15.7%)	4.11 (0.66-25.7)	0.144
History of cancer (vs. no history of cancer)	3 (5.9%)	0.00 (0.00-15.4)	0.305
Sex (male vs. female)	32 (62.7%)	0.50 (0.06-4.20)	0.480
Dyslipidaemia (vs. no dyslipidaemia)	12 (23.5%)	0.73 (0.08-6.58)	1.000
Diabetes Mellitus (vs. no Diabetes Mellitus)	5 (9.8%)	0.00 (0.00-8.94)	1.000
Obesity (vs. no obesity)	9 (17.6%)	1.18 (0.13-10.7)	1.000
Aortic dissection (vs. no aortic dissection)	2 (3.9%)	0.00 (0.00-20.2)	1.000
Previous endograft (vs. no previous endograft)	4 (7.8%)	0.00 (0.00-10.7)	1.000

OR: odds ratio. CI: confidence Interval. ASA: American Society of Anesthesiologists physical status classification. ICHD: ischaemic coronary heart disease. eGFR: estimated glomerular filtration rate (KDIGO).

**Table 5:** Multivariable logistic regression of key baseline characteristics of the study population.

Variable	Adjusted OR	95% CI	p-value
Age (per 1-year increase)	1.07	(0.89-1.39)	0.563
ASA class IV (vs. ASA III)	4.54	(0.21-192.06)	0.352
History of stroke (vs no history of stroke)	30.48	(1.58-1390.36)	0.034
ICHD (vs. none)	3.95	(0.34-95.87)	0.297
eGFR <60 mL/min/1.73 m² (vs ≥60)	2.14	(0.12-70.60)	0.616

OR: odds ratio. CI: confidence Interval. ICHD: ischaemic coronary heart disease. ASA: American Society of Anesthesiologists physical status classification. ICHD: ischaemic coronary heart disease. eGFR: estimated glomerular filtration rate (KDIGO).

The mean number of visceral stents used was 2.6 (range: 1-5) in PMEG-treated patients and 3.8 (range: 2-4) in those treated with t-BRANCH. PMEG devices required a mean of 2.7 fenestrations per patient, whereas t-BRANCH devices did not require fenestrations due to their branched design.

The incidence of postoperative endoleaks was higher in the t-BRANCH group (38.5%) compared with the PMEG group (13.2%). Although most were type II endoleaks, not all cases required immediate reintervention.

## Discussion

This single-centre observational study describes our institution’s experience with the endovascular treatment of complex aortic aneurysms. The results demonstrate low in-hospital mortality and a low incidence of major complications and reinterventions, highlighting the safety and effectiveness of PMEGs and t-BRANCH devices in a highly complex setting. In Latin America, documented experience with endovascular treatment of complex aortic aneurysms remains limited, largely due to restricted access to commercial devices because of their high cost and the limited availability of technical expertise, underscoring the need for regional studies. Recent reports from Argentina have described encouraging outcomes with these devices, supporting the feasibility of these therapies even in the context of structural and logistical constraints ^9^^,^^10^. A report by Gómez et al. from Colombia on several cases treated with fenestrated endografts described a mortality rate of 10%, predominantly among patients with advanced renal disease, and a 10% rate of spinal cord ischaemia. Nevertheless, despite multiple comorbidities that precluded open surgery, follow-up outcomes were satisfactory ^11^.

PMEGs represent a valid alternative for the endovascular treatment of complex aneurysms. Although their use remains heterogeneous, there is a clear association between case volume and outcomes, as demonstrated in the report by O’Donnell et al., where PMEG performance was comparable to that of commercial devices ^12^. Higher procedural volumes are associated with improved outcomes in terms of mortality and complications.

The population in our study consisted predominantly of patients aged around 70 years, mostly men, a pattern consistent with reports from the United States and Europe, where male sex and smoking have been identified as significant risk factors for the development of abdominal aortic aneurysms ^13^^,^^14^. In many cases, these lesions are initially infrarenal; however, in our region, unlike countries with more robust healthcare systems, screening is less rigorous and preventive health culture is limited, contributing to delayed diagnosis.

Regarding comorbidities, hypertension, smoking, and dyslipidaemia predominated, findings consistent with other studies of patients treated with complex endografts in centres across the United States and Western Europe ^6^. The ASA classification was particularly relevant, as most patients classified as ASA IV belonged to the group that experienced mortality, emphasising the importance of optimising perioperative management in this subgroup.

The overall mortality in our series is consistent with that reported in comparable studies, such as the Norwegian multicentre study by Harda *et al.* (9%) ^15^ and the retrospective study by Kölbel *et al.* (8.5% in elective patients) ^13^. In the PMEG subgroup, mortality was 5.3%, slightly higher than that reported in the Zenith trial by Oderich *et al.* (1.5%) and by Starnes et al. (2%) ^16^^,^^17^. These differences may be explained by the fact that those studies included only juxtarenal aneurysms, whereas our cohort also included thoracoabdominal and paravisceral aneurysms. In the t-BRANCH group, early in-hospital mortality was 23.1%, without reaching statistical significance, likely due to greater anatomical complexity and the small sample size in this subgroup. Georgiadis *et al.* reported mortality rates of 3.2% for off-the-shelf devices and 1.1% for PMEGs, confirming the safety of both strategies in elective and urgent settings ^18^. Overall, an early in-hospital mortality rate of 5.3% for PMEGs appears acceptable and comparable to international series, despite the limited number of cases. 

In terms of neurological complications, Juszczak *et al.* reported a paraplegia rate of 1.9% in patients treated with PMEGs, attributed to their spinal cord protection protocol and staged repair with temporary aneurysm sac perfusion ^19^, findings comparable to ours, as our centre also performs staged repair. For t-BRANCH, spinal cord ischaemia remains the main concern. A meta-analysis by Konstantinou *et al.* reported a rate of 12.2% (95% CI: 4.1%-23.2%), with only one case of major stroke (20), figures consistent with our experience.

Regarding reinterventions, the t-BRANCH group showed a rate of 7.7%, similar to that reported in the European meta-analysis (5.7%; 95% CI: 1.7%-11.4%) ^22^, whereas the PMEG group had a rate of 5.3%, lower than the 13.8% reported in a multicentre study of 1,274 patients ^21^, likely reflecting differences in sample size.

Among predictors of mortality, ASA IV physical status (OR = 11.98; 95% CI: 1.46-98.7; p = 0.022) and a history of stroke (OR = 13.07; 95% CI: 1.06-161.5; p = 0.043) were identified as significant factors. Although not statistically significant, coronary artery disease and arterial dysfunction warrant attention, as they may be associated with increased perioperative risk, as demonstrated in the multicentre study by Tsilimparis *et al.*, which identified peripheral arterial disease and reduced glomerular filtration rate as independent predictors of major adverse events ^21^.

Our findings are comparable with those of the most recent multicentre study from the International Multicenter Aortic Research Group, which included 27 centres and 3,634 patients treated for thoracoabdominal aortic aneurysms with fenestrated endografts, reporting a 5% in-hospital mortality rate and a significantly higher rate of adverse events in low-volume centres (fewer than 11 cases) compared with high-volume centres (33% vs. 20%; p<0.001). Similar predictors of mortality were identified, including age, chronic kidney disease, ASA class ≥3, prior aortic repair, symptomatic or ruptured aneurysm, and Crawford types I-III ^22^. 

With regard to reinterventions, although they were more frequent in the t-BRANCH group, no major complications were observed after correction, except for type II endoleaks managed on an outpatient basis, confirming the effectiveness of both procedures.

The main limitations of this study include its single-centre design and small sample size, particularly in the off-the-shelf device subgroup, which limits statistical power to detect significant differences between groups. In addition, the lack of randomisation and device selection based on availability and anatomical characteristics may introduce selection bias.

In conclusion, endovascular repair of complex thoracoabdominal and paravisceral aortic aneurysms using PMEGs and t-BRANCH devices yields comparable outcomes in terms of mortality and major postoperative complications, with a low rate of reintervention. Although outcomes tended to favour PMEGs, with lower mortality and reintervention rates, albeit without statistical significance, both devices proved effective, even in urgent settings. These findings underscore the need for multicentre series and regional registries to further validate these results.

**Figure 1:**
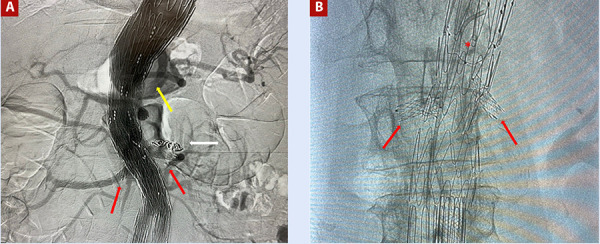
Image **A** shows a branched endograft to the coeliac trunk (yellow arrow), superior mesenteric artery, and renal arteries (red arrows), with additional embolisation of the left polar artery (white arrow). Image **B** demonstrates a surgeon-modified endograft with a scallop for the superior mesenteric artery (red asterisk) and two fenestrations for the renal arteries (red arrows).
